# Modified Poly(vinylidene fluoride) by Diethylenetriamine as a Supported Anion Exchange Membrane for Lithium Salt Concentration by Hybrid Capacitive Deionization

**DOI:** 10.3390/membranes12020103

**Published:** 2022-01-18

**Authors:** Anna Siekierka, Marek Bryjak

**Affiliations:** Department of Process Engineering and Technology of Polymeric and Carbon Materials, Wroclaw University of Science and Technology, Wyb. Wyspianskiego 27, 50-370 Wroclaw, Poland; marek.bryjak@pwr.edu.pl

**Keywords:** poly(vinylidene fluoride), diethylenetriamine, lithium salt concentration, hybrid capacitive deionization

## Abstract

This paper shows the investigation for the optimal anion exchange membranes (AEM) supporting the desorption step of the HCDI process. The chemical modification of PVDF by diethylene triamine created the AEM. To confirm the ion-exchange character of materials, the chemical analysis with FTIR, SEM, surface energetics, and transportation analysis were applied. Next, the investigated membranes were applied for the sorption and desorption of lithium chloride. The specific sorptive parameters were higher according to the incorporation of the nitrogen groups into polymeric chains. Considering the desorption efficiency, membranes modified by four days were selected for further evaluation. The application in the HCDI process allowed reaching the desorption efficiency at 90%. The system composed of PVDF-DETA4 membrane was suitable for sorption 30 mg/g of salt. By applying the PVDF-DETA4 membrane, it is possible to concentrate LiCl with four factors. The anion exchange character of the developed membrane was confirmed by adsorption kinetics and isotherms of chlorides, nitrates, sodium, and lithium. The prepared membrane could be considered a perspective material suitable for concentration salt with electro-driven technologies for the above reasons.

## 1. Introduction

The hybrid capacitive deionization (HCDI) is an electro-membrane process dedicated to selective removal species like ions in the minority at aqueous solutions [1]. The cell of HCDI is built from selective cathode material like lithium spinel-type sorbent and a composite anode made with activated carbon coated by anion exchange membrane [2]. Typically, as a cathodic material the sorbents like lithium–manganese–titanium oxide (LMTO) [3], lithium manganese iron oxide (LMFO) [4], nickel hexacyanoferrate (NiHCF) [5], manganese oxides [6], sodium manganese oxide (NMO) [7], lithium manganese oxides (LMO) or made with activated carbon modified by inorganic oxides [8], silver [9] or conductive polymers [10] are used. On the other hand, the composite anode electrode is needed to reduce the re-sorption of co-ions during the discharging step [11]. As a result, ions adsorbed are being removed from the selective electrode and, at the same time, are being captured by the counter electrode.

In consequence, the desorption process is challenging to control. Therefore, the anion exchange membrane should be applied to prevent this undesirable phenomenon. Hence, the ion exchange membranes have the critical function of controlling the efficiency of ions recovery. For lithium capturing, the primary cell of HCDI is comprised of lithium selective adsorbent as the cathode and activated carbon electrode wrapped with the anion-exchange membrane as the composite anode.

Poly(vinylidene fluoride), PVDF, is a highly versatile polymer with an excellent balance between its comprehensive properties and numerous applications. It has many advantages, such as low permittivity, comprehensive frequency response, flexibility, low resistance loss, easy fabrication, biocompatibility, and cost-effectiveness [12]. However, the PVDF has to be modified as the functional membrane for electro membrane applications. Using some chemical reactions, it is possible to obtain a wide range of functional membranes containing functional groups that enhance the antifouling phenomenon and have pH-sensitivity or ion-selectivity character [13]. Moreover, the PVDF polymer has a powerful ability to substitute fluorine atoms into functional groups. Previously, the PVDF was modified by ethylenediamine to produce an anion exchange membrane (AEM). The modification was successful, and AEM with -NH_2_ anion exchange groups was developed. Consequently, the modification of PVDF by an active agent with different amine groups should be investigated to see the differences between EDA and DETA. The main objective of this study was to obtain a PVDF membrane with good mechanical properties, chemically stable, and a high concentration of anion-groups onto its surface. It was done by reacting PVDF with diethylenetriamine (DETA). According to the chemical structure of poly(vinylidene fluoride), the addition of DETA causes two reactions: (1) direct crosslinking via intrachain dehydrofluorization and (2) indirect crosslinking via intrachain dehydrofluorization [14], followed by the Michael addition [15]. In this paper, the method of preparation PVDF-DETA membranes for lithium salt concentration via the HCDI process has been shown. The main aim of the work was to find the synthetic paths of PVDF modification leading to obtaining anion exchange membrane. The second objective was to evaluate the PVDF-DETA for lithium extraction and concentration in the desorption step. In addition, the investigation of adsorption kinetics and isotherms of chloride anions transportation through membranes was conducted.

## 2. Materials and Methods

### 2.1. Materials

Poly(vinylidene fluoride) (PVDF) with a molecular weight of 180,000 g/mol and diethylenetriamine (DETA) were supplied by Sigma-Aldrich. N, N-dimethylformamide (DMF), chloric acid, lithium chloride, sodium hydroxide, and ethanol (96%) were purchased from Avantor Performance Materials, Poland S.A. Deionized water (DI) was delivered from RO Water Purification Systems Millipore (14.4 MΩ/cm^2^).

### 2.2. Membrane Fabrication

The PVDF was dissolved in DMF (96 h at room temperature) to obtain a homogeneous solution with 15% wt. of PVDF. Then, the films of 0.20 ± 0.05 mm of thickness were cast into a glass plate and dried overnight in a vacuum dryer. Next, the films were immersed in DETA (100% concentration, 20 mL of volume) and kept for 1, 2, and 4 days. The prepared anion exchange membranes were encoded PVDF-DETA1, PVDF-DETA2, and PVDF-DETA4. After modification, membranes were rinsed with DI water and ethanol and kept in 40% wt. an aqueous solution of ethanol. 

### 2.3. AEM Characterization 

#### 2.3.1. Scanning Electron Microscope (SEM)

The scanning electron microscope (SEM, Tescan Vega3 SB) was used to determine the morphology of obtained PVDF-EDA membranes. All samples were gold-coated with a 7 nm thick layer.

#### 2.3.2. Fourier Transform Infrared Spectroscopy

Fourier transform infrared spectroscopy in the range of 4000–400 cm^−1^ (Vertex 70 vacuum spectrometer equipped with the horizontal ATR device) was used to identify the presence of amine groups into polymer films. For each analysis, 64 scans were collected. In addition, the FTIR spectra were recorded for dry membranes.

#### 2.3.3. Surface Energetics

Contact angles of such probing liquids as water, formamide, and diiodomethane were measured at 25 °C through goniometer PG-X (Fibro System AB). The results were given as the average of 10 independent measurements for each liquid.

#### 2.3.4. The Analytical Section

##### Water Uptake

Water uptake $W_{H_{2}O}$ [$\frac{g_{H_{2}O}}{g}\;]$ was determined from Equation (1):

(1) $$W_{H_{2}O}=\frac{\left(m_{w}-m_{d}\right)}{m_{d}}$$

where *m_w_* is the weight of swollen membrane and *m_d_* is the weight of the dry membrane.

##### Ion Exchange Capacity

Ion-exchange capacity (*Z_IEC_*) was estimated employing the acid-base titration method. Membrane sample was placed into an Erlenmeyer flask, and 50 mL of 0.1 M NaOH solution was added. The membrane was kept in the solution for 24 h at room temperature. After that time, 10 mL of solution was taken and titrated with 0.1 M HCl solution. The ion-exchange capacity *Z_IEC_* $\left[\frac{mmol}{/g}\right]\;$ was calculated from Equation (2):

(2) $$Z_{IEC}=\frac{\left(c_{b}\cdot v_{b}-c_{a}\cdot v_{a}\right)\;}{m_{d}}$$

where *c_b_* and *c_a_* are the molar concentrations of the NaOH and HCl, respectively, *v_b_* is the volume of NaOH taken for the titration, *v_a_* is the volume of HCl used for the titration of the NaOH solution, and md is the weight of the dry membrane. 

##### Nitrogen Content

Kjeldahl’s method determined nitrogen content (*Z_N_*) after mineralizing the sample (about 200 mg) in concentrated sulfuric acid with copper and potassium sulfates.

##### Diffusion Dialysis

The DD process was carried out in a two-compartment cell divided by a flat membrane with the active area at 4.91 cm^2^. Each compartment was stirred by a magnetic bar rotating at 200 rpm. The feeding (0.1 M HCl) and stripping (DI water) solutions, 35 mL each, were placed on both sides of the membrane. The experiments were conducted for 30 min, and flux, *J* (mole/m^2^ s), was calculated according to Equation (3):

(3) $$J=-\frac{V}{S}\cdot \frac{dC}{dt}\;$$

where *V* is the volume of the compartment, *S* is the membrane’s effective area, and *t* is the time of DD. 

Fick’s first law expresses the mass transport through anion exchange membranes and is given by *J* = κΔ*C*, where Δ*C* (mole/dm^3^) is the concentration gradient, and *k* (m/s) is the rate mass transfer coefficient. Finally, after integration, the following expression was used:

(4) $$\ln\left(\frac{C}{C_{o}}\right)=-k\left(\frac{S}{V}\right)t$$

where *C_o_* (mole/dm^3^) is the initial concentration of HCl. 

#### 2.3.5. HCDI Process

##### HCDI Configuration

**Figure 1:**
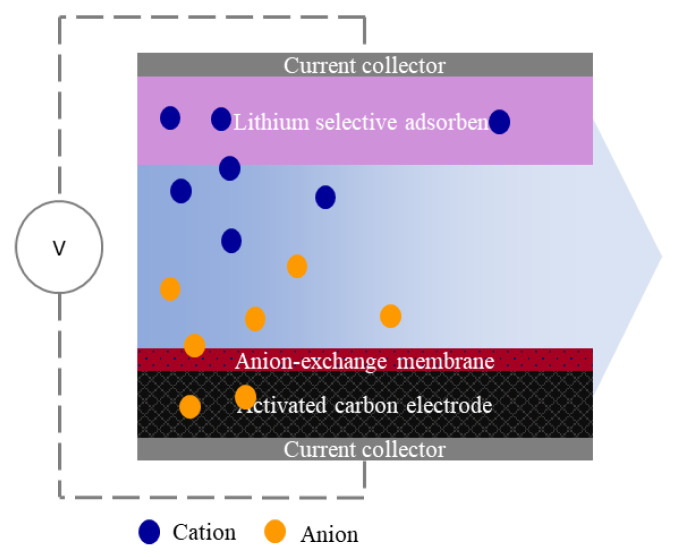
Scheme of HCDI cell.

A laboratory electrodialysis FT-ED-100-4, FumaTech, was used to study anion-exchange membrane performances. The stack was composed of two parallel electrodes divided by a polymeric spacer of 200 µm thickness. As a cathode, the lithium-manganese-titanium oxide (LMTO) were mounted. The anode comprised an electrode made of activated carbon, YP-50F, and covered by PVC-DETA anion-exchange membrane. The general scheme of the HCDI cell is presented in Figure 1. The electrolyzer was biased by Multi-Range Programmable DC Power Supplies BK Precision 9201 and controlled by DC Electronic Load BK Precision 8601. The tests were conducted under constant voltage (CV). In addition, the CX-601 multimeter was applied to monitor the feed solution conductivity, pH, and temperature. The used parameters of the HCDI process are shown in Table 1. 

According to Mohr protocol, the titration method determined the concentration of chlorides [4]. The Kjeldahl procedure determined the concentration of nitrates to analyze nitrogen in samples [16]. Finally, ion-selective electrodes were determined the sodium and lithium concentrations from Elemetron S.A., Poland, and Metler-Toledo, Poland. 

##### HCDI Calculations

Fundamental factors for the capacitive deionization process, like salt adsorption capacity (SAC), salt desorption capacity (SDC), average salt adsorption rate (ASAR), and average salt desorption rate (ASDR), were calculated. The SAC determines the adsorbed salt (represented by a single ion) per gram of applied active material (90% of total electrode weight), while SDC represents the desorbed salt amount. When the amount of adsorbed/desorbed salt was normalized to the processing time, it showed the average salt adsorption/desorption rate (ASAR/ASDR), a valuable metric for the process description. The SAC, ASAR, and SDC, ASDR indicate the general adsorption/desorption capacity and rate delivered from initial and final concentrations of ions or online according to the time step. Furthermore, the adsorption and desorption operations were also performed without an external electrical field. This case shows how the electrical potential/current influenced charge/current efficiency and adsorption/desorption behaviors. 

The simple RC (resistance–capacitor) circuit with measurements of current change was applied to analyze the energy consumption and calculate the system capacity. First, the energy consumption (EC) was computed from numerical integration of the current versus time relationship and voltage. Then, the following metrics of energy normalized adsorption/desorption of salt (ENAS and ENDS) in gram units per Joule of energy were calculated. The next factor describing HCDI was electrical work in Wh per gram of adsorbed/desorbed salt, and it was defined as a ratio of charge flow by the system during the adsorption/desorption step with an electrical potential between electrodes divided by the mass of adsorbed/desorbed salt.

#### 2.3.6. Theoretical Background of Anion Transportation

The pseudo-first-order [17], pseudo-second-order [18], Weber–Morris intraparticle diffusion [19] and Elovich models [20] were applied for the investigation of the adsorption kinetics and transportation of chloride anions through developed anion exchange membranes. On the other hand, the Temkin [21] and Harkins-Jura [22] models were applied for adsorption isotherms.

##### Pseudo-First-Order Kinetic Model

The pseudo-first-order (PFO) rate expression of Lagergren and Annadurai and Krishnan in linear form is given as:

(5) $$\log\left(q_{e}-q\right)=\log\left(q_{e}\right)-\frac{k_{1}t}{2.303}$$

With *q* the amount of adsorbed solute, *q_e_* its value at equilibrium, *k*_1_ the pseudo-first-order rate constant and *t* the time. The pseudo-first-order kinetic constant and the theoretical *q_e_* based on pseudo-first-order kinetics can be obtained from the plot of log(*q_e_* − *q*) versus *t*. The equilibrium adsorption density *q_e_* is required to fit the data, but in many cases, *q_e_* remains unknown due to slow adsorption processes. In addition, in many cases, the first-order equation of Lagergren does not fit well to the whole range of contact time and is generally applicable over the initial stage of the adsorption processes.

##### Pseudo-Second-Order Kinetic Model

The pseudo-second-order (PSO) kinetics model can be rewritten in linear form as:

(6) $$\frac{t}{q}=\frac{1}{k_{2}q_{e}^{2}}+\frac{1}{q_{e}}t$$

The PSO kinetic constant and the theoretical *q_e_* by a type 1 PSO expression can be calculated from the plots of *t*/*q* versus *t*. The PSO model is more likely to predict the behavior over the whole range of adsorption and agrees with chemical sorption being the rate-controlling step [23] which may involve valency forces through sharing or exchange of electrons between ions and adsorbent.

##### Weber–Morris Intraparticle Diffusion Model

The intra-particle diffusion model based on the theory proposed by Weber and Morris [24] was used to identify the diffusion mechanism. According to this theory, the adsorbate uptake *q_e_* varies almost proportionally with the square root of the contact time, *t*½ rather than t. this model is given as:

(7) $$q_{t}=k_{id}\sqrt{t}+C$$

*K_ID_* is the intraparticle diffusion constant (mg/g min^0.5^), and the intercept (*C*) reflects the boundary layer effect. The *k_id_* values were calculated from slopes (*k_id_*) of the plots of *q_e_* vs. *t*^0.5^ [25]. The intraparticle diffusion model describes adsorption processes, where the rate of adsorption depends on the speed at which adsorbate diffuses towards adsorbent (i.e., the process is diffusion-controlled) [26].

##### Elovich Model

The Elovich equation is given as follows [27]:

(8) $$q_{t}=\frac{1}{\beta }\ln\left(\alpha \beta \right)+\frac{1}{\beta }\ln\left(t\right)$$

where *α* (mg/g min) is the initial sorption rate, and the parameter *β* (g/mg) is related to the extent of surface coverage and activation energy for chemisorption. The kinetic results will be linear on a *q_t_* versus *ln*(*t*) plot. It was suggested that diffusion accounted for the Elovich kinetics pattern; conformation to this equation alone might be taken as evidence that the rate-determining step is diffusion in nature and that this equation should apply at conditions where desorption rate can be neglected.

##### Temkin Model

The derivation of Temkin adsorption isotherm assumes that the fall in the heat of adsorption is linear. This sorption isotherm contains a factor that explicitly considers the interaction between adsorbate and adsorbent. Due to interactions between adsorbent and adsorbate, the heat of sorption of adsorbing ions in the layer decreases linearly with the coverage of the adsorbent surface. Temkin isotherm takes into account the occupation of high energetic sites at first. The linearized form of the isotherm is [27]:

(9) $$q_{e}=\frac{RT}{b_{T}}lnA_{T}+\frac{RT}{b_{T}}lnC_{e}$$

where *A_T_* (L/g) and *b_T_* (kJ/mol) are Temkin constants related to equilibrium binding constant (L/mol), which are related to the maximum binding energy and heat of adsorption, respectively. These constants were calculated from the slope and intercept of the plot *q_e_* vs. *lnC_e_*.

##### Harkins–Jura Model

The multilayer adsorption and the existence of the heterogeneous pore distribution in the surface of the adsorbents are mainly described by Harkins–Jura isotherm model [28], which is expressed as:

(10) $$\frac{1}{q_{e}^{2}}=\frac{B_{HJ}}{A_{HJ}}-\frac{1}{A_{HJ}}\log C_{e}\;$$

*B_HJ_* and *A_HJ_* are the Harkins–Jura constants; the value of *B_HJ_* and *A_HJ_* can be determined from the slope and intercept of the plot of 1/*q_e_*^2^ vs. log *C_e_*, respectively [28].

## 3. Results and Discussion

### 3.1. Membrane Characterization

#### 3.1.1. Membrane Morphology

**Figure 2:**
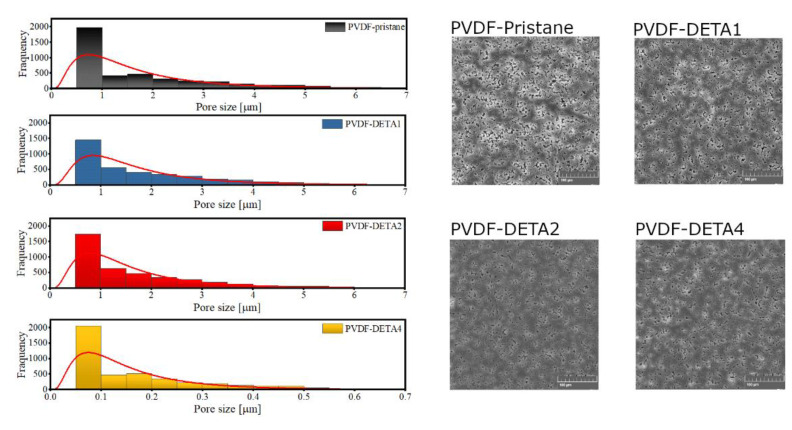
SEM images and pore size distribution for investigated anion exchange membranes.

In Figure 2, the surface of PVDF-DETA membranes is presented. It could be noted that the morphology has been changed during the amination time. The aggressive environment (pK_b_ ~ 11) could affect the polymer film in two ways. First, DETA damages the polymeric film abides the polymeric surface or accumulates with plugging pores. In the case of PVDF modification, the membrane shows smaller pores than for pristine film. Therefore, it could be suggested that on the PVDF surface, some aggregates were deposited. After 1 and 2 days of modification, Pristane PVDF and membrane have a pore size between 0.5 to 6 nm. Only membrane immersed for four days exhibits pore size under 1 nm. 

#### 3.1.2. Membrane Chemistry

**Figure 3:**
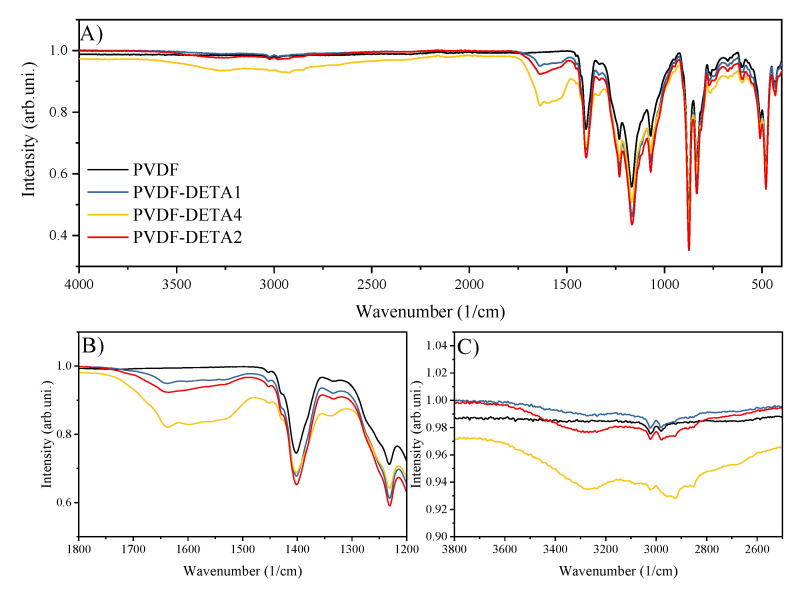
FTIR spectra for investigated AEMs.(**A**) FTIR spectra in whole range; (**B**) approximation of FTIR in range 1800–1200 cm^−1^; (**C**) approximation of FTIR in range 3800–2500cm ^−1^.

The FTIR spectra for pristane and modified PVDF-DETA membranes are presented in Figure 3. At 11, the adsorption bands could be assigned to the secondary amine groups (Figure 3B,C). However, the CH_2_ bending mode expected to appear at 1450 cm^−1^ was found at 1440 cm^−1^. Therefore, the exposition of PVDF films to DETA resulted in dehydrofluorination and crosslinking of polymer matrix [29]. 

**Figure 4:**
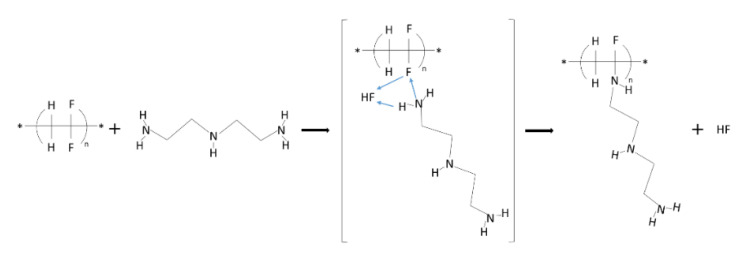
Mechanism of nucleophilic addition diethylenetriamine into poly(vinylidene fluoride) chain.

On that base, the expected mechanism of the PVDF modification followed the Michael reaction [15]. During the reaction, some unsaturated bonds were created that caused the addition of diethylenetriamine. The reaction between PVDF and DETA altered surface character [29]. The mechanism of wrapping diethylene triamine into PVDF chains is presented in Figure 4. 

**Figure 5:**
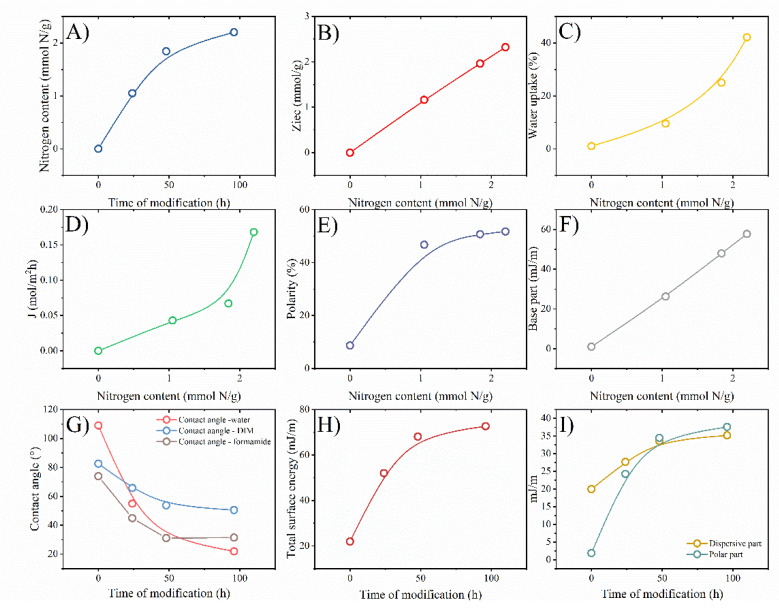
Chemical characterization of modified series of PVDF membranes. Effect of time modification on nitrogen content (**A**), effect of nitrogen content on ion-exchange capacity (**B**), nitrogen content vs. water uptake (**C**), nitrogen content vs. flux (**D**), nitrogen content vs. polarity (**E**), nitrogen content vs. the base component of the total energy surface (**F**) time modification vs. contact angles (**G**), time modification vs. total surface energy (**H**) time modification vs. dispersive and polar component (**I**).

Their nitrogen content, ion exchange capacity, water uptake, and surface energetics were investigated to characterize the aminated PVDF membranes. Figure 5A can show the changes in nitrogen content over the modification time. It was found that with the progress of modification, the nitrogen raised linearly in the range between 1 to 3 days. On the fourth day of modification, the nitrogen content also increased slower. Its growth changed from ~1 mmol of N/g per day for the first three days and reached a value of ~0.4 mmol of N/g on the last day. In Figure 5B, the correlation between ion exchange capacity and nitrogen content in PVDF films. The *Z_IEC_* is linearly dependent on the nitrogen content that resulted in NH_3_^+^ functionalities. The nitrogen content also affected water uptake (Figure 5C) so that it grew exponentially with the increase of nitrogen amounts. This phenomenon is associated with the hydration of amine groups. Figure 5D the correlation of nitrogen content and Cl-flux. The flux of chloride anions increased exponentially with nitrogen content. It seems this is associated with anion-exchange groups’ presence in the modified PVDF films. Hence, the modification of PVDF by incorporating anion exchange groups -NH_3_^+^ from DETA turned PVDF films into anion exchange membranes (Figure 4). 

#### 3.1.3. Surface Energetics

The contact angles of DI water, DIM, and FA were performed to characterize the surface energy and its components. Based on the results shown in Figure 5G, the polar component (Figure 5F), dispersive component (Figure 5I), and total surface energy (Figure 5H) were calculated. Wu’s protocol was applied to calculate dispersive and polar parts, and the van Oss, Chaudhury, and Good procedure served to get the base component. 

The polarity was calculated as a share of the polar component to total surface energy. According to obtained data, the polarity reached a level ~50% after one day of modification, and its value was stable over the additional time. This fact is associated with a similar tendency to change total surface energy and its polar and dispersive components. Their values were changed with the modification time with the plateau region after the four days. Hence, further incorporation of amines into the PVDF chain did not affect either total surface energy, polar or dispersive components, or polarity. 

In summary, the PVDF films were successfully modified by DETA according to Michael’s reaction. As a result, the properties of obtained membranes were directly associated with the nitrogen content. 

### 3.2. The Concentration of Lithium Salt by HCDI

#### 3.2.1. Selection of PVDF-DETA Membrane

**Figure 6:**
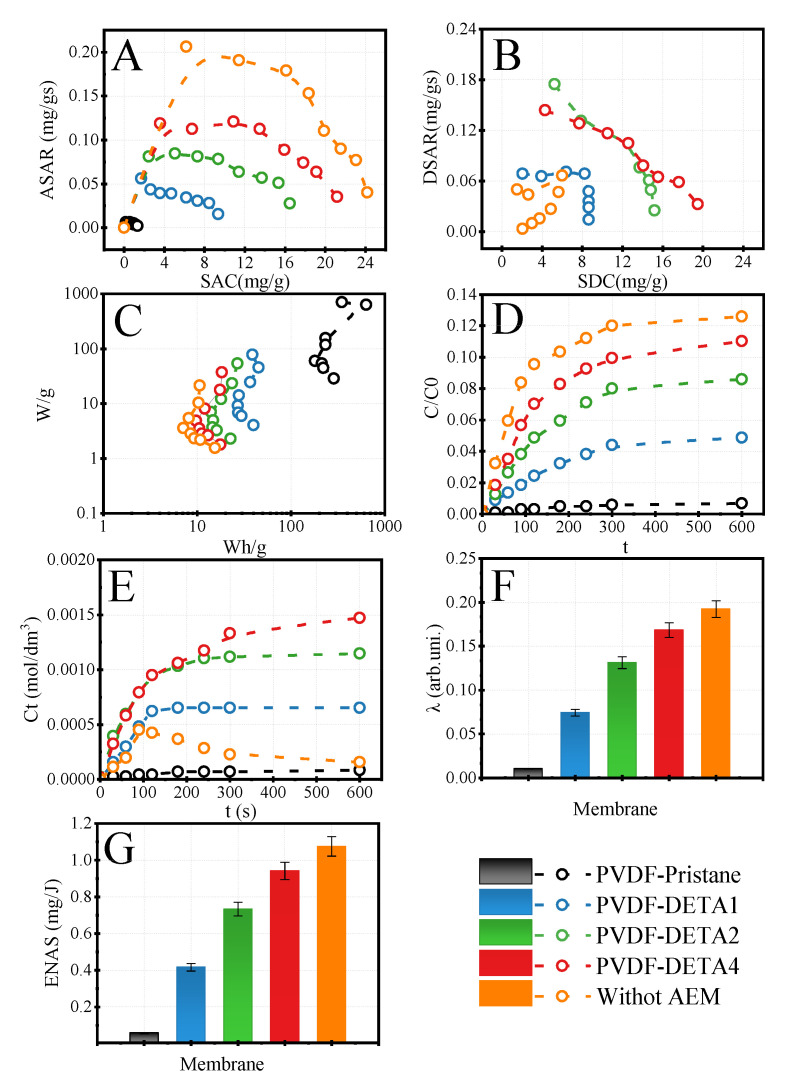
Modified Ragone plots for adsorption (**A**) and desorption (**B**) of LiCl salt, classical Ragone plot for adsorption (**C**), the changing of the relative concentration of lithium chloride during adsorption (**D**), lithium chloride concentration released (**E**) during desorption, current efficiency (**F**) and ENAS (**G**) for adsorption steps for investigated PVDF membranes. C_LiCl,feed_ = 10 mM, CV_ads_ = 1 V, CV_des_ = 0 V t_ads_ = t_des_ = 10 min, V_feed_ = 0.1 dm^3^.

The next step for evaluating PVDF-DETA membranes was the selection of the best AEMs for concentration LiCl in the HCDI process. The cell of HCDI was comprised of LMTO active cathode material and a combined counter electrode made of activated carbon active material covered by the investigated anion-exchange membranes. All of the experiments were performed under the same conditions. The SAC values of the investigated membranes were compared with the system without AEM. The data are shown in Figure 6. The significant changes of SAC and ASAR were observed for various types of applied membranes. The minimal SAC was reached by configuration with PVDF-Pristane films. This fact was expected as the unmodified PVDF films had no ion-exchange groups. The SAC of 2 mg/g suggests that even with the use of LMTO material dedicated for lithium sorption, the critical parameter was connected to anion exchange membranes that controlled the growth of resistance in the HCDI system. Thus, the AEM membranes affected the system resistance, which decreased the sorption efficiency of HCDI. PVDF membranes exhibit the linear relationship between SAC values and ion exchange capacity, expressed as nitrogen content. The highest SAC value was determined for PVDF-DETA4. 

Despite the HCDI without AEM membranes having the highest sorption capacity, desorption of accumulated salts was a complex phenomenon. The data is presented in Figure 6B, where the modified Ragone plot compares the desorption ability of the system that, without AEMs, initially desorbed salt performed the desorption, but after ~90 s, the backstream of released ions was observed. This phenomenon is also found in Figure 6E. In the case of the presence of the AEM membranes, the salt concentration in the desorption step raised slowly when the PVDF membranes blocked the co-ions entrance to counter electrodes. The opposite situation is observed in the configuration without ion-exchange membranes. Here, the re-sorption of cations into a nonblocked counter electrode was observed. The effect disappeared when the PVDF membranes were attached to the electrode. The highest impact on blocking the re-sorption of counter ions showed the PVDF-DETA4 membrane. 

The energetic aspects of sorption are presented in Figure 6C. The Ragone plot presents the power and energy consumption during the charging step of the HCDI systems. The highest energy consumption was detected for the system with unmodified membranes, creating a non-conductive barrier. The highest current efficiency was achieved for HCDI equipped with AEM membranes. However, the PVDF-DETA4 configuration exhibited an 11% lower value and reached 0.17. The rest membranes had higher current efficiency than pristine PVDF that could be associated with ion exchange capacity and promotion of sorption phenomenon. The last parameter, ENAS, shows how many grams of salt could be absorbed per one Joule of energy. In this case, the highest ENAS was also detected for the system without membrane, viz. 1.1 mg/J. The configuration with PVDF-DETA4 reached 0.9 mg/J. 

#### 3.2.2. Selection of HCDI Voltage Conditions

**Figure 7:**
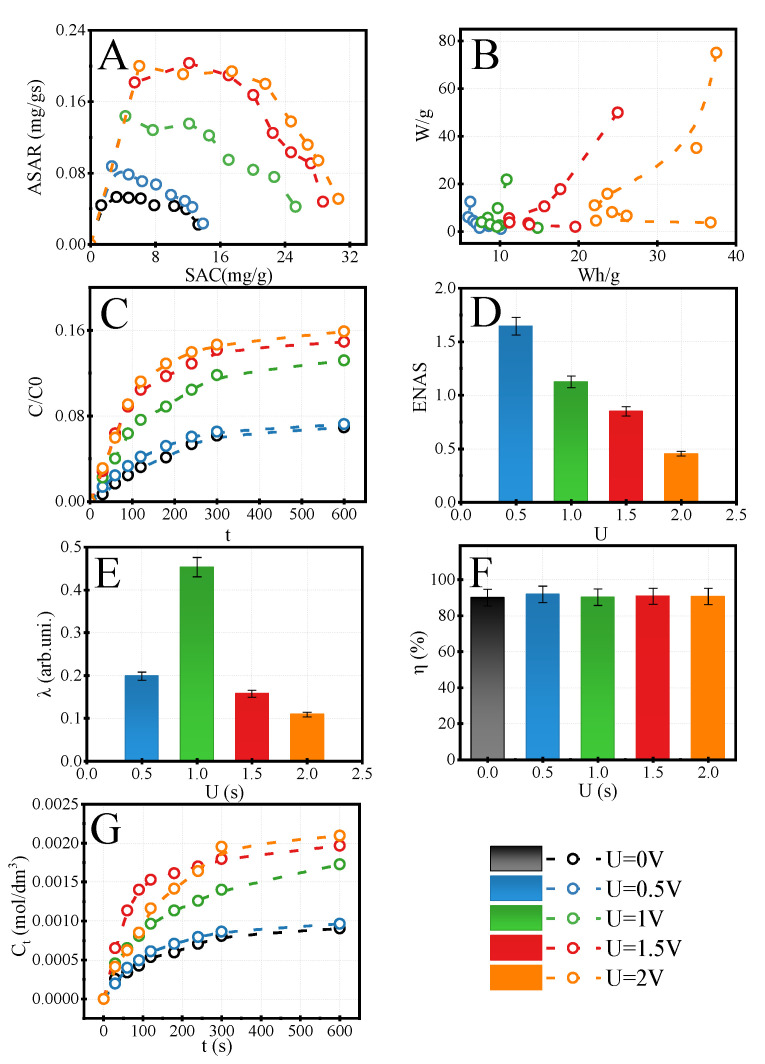
Modified Ragone plots for adsorption (**A**) of LiCl salt, classical Ragone plot for adsorption (**B**), The changing of relative concentration of lithium chloride during adsorption (**C**), ENAS (**D**) current efficiency (**E**) desorption efficiency (**F**), and lithium chloride concentration released during desorption (**G**) for PVDF-DETA4 over various voltage in CV mode. C_LiCl,feed_ = 10 mM, t_ads_ = t_des_ = 10 min, V_feed_ = 0.1 dm^3^, CV_des_ = 0 V.

From the above data, the most promising AEM membrane for the HCDI process is PVDF-DETA4. However, the optimal electrical conditions related to the charging step should be found. We evaluated a different external voltage charge in constant voltage to do it. The effects on SAC, energetical factors and kinetics of accumulation LiCl salts are presented in Figure 7. With increasing external voltage, the SAC value (Figure 7A) is raised from ~14 mg/g for U = 0.0 V to ~30 mg/g for U = 2.0 V and reached twice higher sorption capacity than in configuration without external electrical fields. The relative sorption (Figure 7C) increased from 0.08 mol/dm^3^ to 0.16 mol/dm^3^. The highest growth of accumulation LiCl salt was achieved from U = 1.0 V to U = 2.0 V, where the sorption changed by 17%. The energetical factors at the classical Ragone plot show that the highest external applied voltage consumed the highest energy, and the SAC value took the highest value at maximum U. The differences between U = 0.5 V and U = 2.0 V were about ~300%. 

A similar tendency is visible in the ENAS parameter (Figure 7D), where the effectiveness decreased over three times from 1.5 mg/J to 0.5 mg/J for U = 0.5 V and U = 2.0 V, respectively. The nonlinear relationship was visible in the current efficiency (Figure 7E). The highest value was observed for U = 1.0 V. This fact is related to the relatively high sorption in U = 1.0 V and lower energetics requirements. The desorption efficiency (η) was the next critical studied parameter (Figure 7F). Here, the η got the same value for each electrical mode (~90%). This is connected with the ability of PVDF-DETA4 to stop the resorption of co-ions during the desorption step. The concentration of salt raised in desorption flux according to SAC tendency (Figure 7G).

#### 3.2.3. The Concentration of LiCl by HCDI

**Figure 8:**
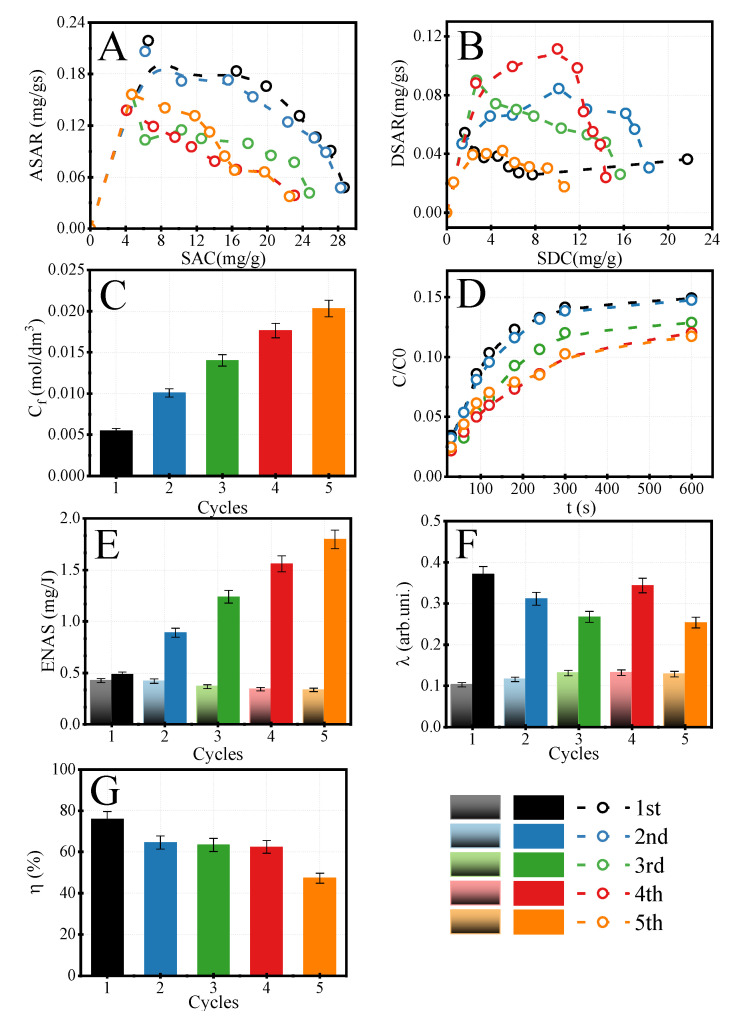
Modified Ragone plots for adsorption (**A**) and desorption (**B**) of LiCl salt, classical Ragone plot for adsorption (**C**), The changing of the relative concentration of lithium chloride during adsorption (**D**), ENAS (**E**), current efficiency (**F**), and desorption efficiency (**G**) for PVDF-DETA4 at U = 2 V (CV mode) for cycles of lithium chloride concentration. C_LiCl,feed_ = 10 mM, t_ads_ = t_des_ = 10 min, V_feed_ = 0.1 dm^3^, CV_des_ = 2.0 V. Two-colored bares are related to adsorption, one-colored bars are related to desorption.

The last investigated issue was to see a chance to concentrate LiCl salt in the desorption step when the discharging process was performed against the concentration gradient. In this case, the five cycles of concentration of LiCl were carried out. The data are presented in Figure 8. Over five sorption cycles, the SAC was decreased from 29 mg/g to 23 mg/g, making a 20% reduction of initial SAC. This is related to limited desorption efficiency and decreasing SDC. The SDC fell from 22 mg/g to 11 mg/g in the first and fifth cycles. This phenomenon is shown in Figure 8C, where the final concentrations of LiCl are presented. The concentration raised from 0.005 mol/dm^3^ to 0.02 mol/dm^3^. Hence, the concentration of LiCl increased four times concerning the initial one. 

The further steps were not equal and were related to the limited sorption and desorption efficiency. The desorption efficiency decreased for further cycles of LiCl concentrating reduced by 30% (Figure 8G). In the case of the ENAS parameter, their values for adsorption (two-colored bares) show a decreased tendency. This fact is directly connected with a decrease in SAC. However, the ENAS for desorption was raised and reached over three times higher values. This showed the possibility to concentrate salt by HCDI. The last parameter, current efficiency, was stable for the adsorption process and reached 0.1 to 0.13 for the first and fifth cycles. 

#### 3.2.4. Comparison with Other Techniques

To complete the analysis of concentration lithium salt by membrane techniques, the comparison investigated PVDF-DETA membrane employed in HCDI with other membranes process is necessary. The Comparison with typical techniques for concentrating compounds is presented in Table 2. The forward osmosis (FO) with NaCl and MgCl_2_ as draw solution for lithium concentration from brines was applied. Here, the commercial membrane from cellulose triacetate (CTA) was used. A Li concentration of 12 g/L was achieved after 30 h, five times higher than the initial feed solution [30]. Next, research under FO was conducted on the application of composite PVDF membrane for lithium concentration. FO is utilized to concentrate LiCl in the solution from 36 to 175 g L^−1^ with an average water transfer rate of 0.450 L m^−2^ h^−1^ (LMH) at 85 °C [31]. Another idea comes from R. Wang team. They applied pervaporation as a potential process for lithium concentration. A specific PVDF composite with graphite oxide (GO) membrane was obtained. Investigated membrane increased lithium concentration from 0.3 to 1.27 g/L (73% feed volume reduction) [32]. In addition, the dialysis process was evaluated as a powerful tool for Li concentration. Parades et al. developed a polymer inclusion membrane composed of cellulose triacetate (CTA) and the carriers LIX-54-100 and Cyanex 923 and applied it for Li concentration. This membrane successfully extracted and concentrated lithium from natural seawater samples. The showed processes are compared with investigated PVDF-DETA4 working in the HCDI process. Despite that, the ratio of concentrating Li during forward osmosis, pervaporation, dialysis, and hybrid capacitive deionization was at the same range between 385 to 700, the rate of concentration for PVDF-DETA4 in HCDI was highest as achieved 0.53 g/h. 

#### 3.2.5. Transportation Phenomenon 

**Figure 9:**
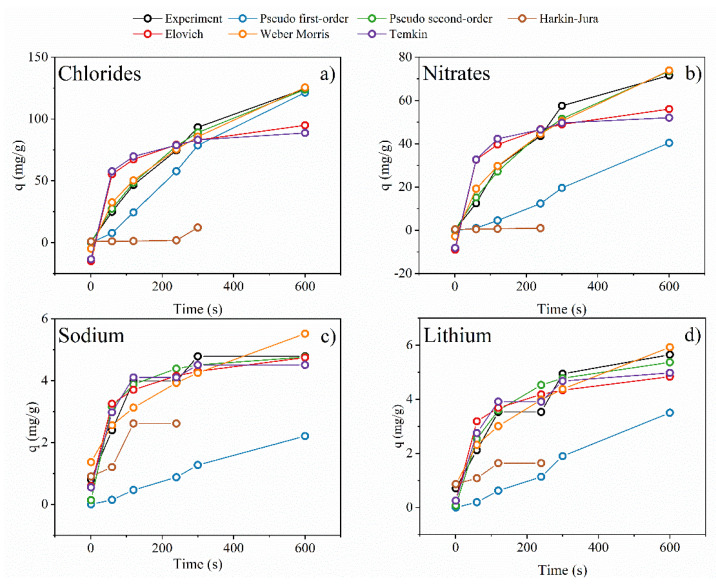
Kinetics and isotherms models for PVDF-DETA4 for (**a**) chlorides, (**b**) nitrates, (**c**) sodium, and (**d**) lithium. Mass of active membrane 0.114 g, initial concentration of each ions 0.1 g/L.

The adsorption kinetics and isotherms are fundamental in explaining the interactives between solutes and membranes and are crucial in designing the ion-exchange behavior of membranes. Considering the ion exchange nature of investigated membranes, comparing anions and cations transportation across the membrane is a critical parameter that will define transported spices’ preferences. According to the nucleophilic substitution mechanism of fluoric atoms by diethylene triamine, primary and secondary amines’ appearance into anion exchange membrane is visible. To confirm the anion-exchange character of the investigated membrane, the PVDF-DETA4 was chosen for transportation chlorides, nitrates as anions representants and sodium and lithium as cations representants. In order to investigate the kinetics of sorption, pseudo-first-order, pseudo-second-order equations, and Weber–Morris and Elovich models were applied. To evaluate the adsorption isotherms, the Temkin and Harkin-Jura models were chosen. The experimental data with fitting models are presented in Figure 9. Additionally, the calculated parameters of PFO, PSO, WM, Elovich, Temkin and Harkin-Jura models are summarized in Table 3. The PFO model fits well for chloride and nitrate transportation reached 121.4 and 40.4 mg/g with R^2^ at 0.963 and 0.943, respectively. During sodium and lithium, PVDF-DETA4 transported only 2.20 and 3.50 with R^2^ at 0.658 and 0.888, respectively. A good correlation with the PFO model during chlorides and nitrates shows a reversible reaction between anions and anion-exchange groups like primary and secondary amines [35]. Based on these facts, the permselectivity of Cl^−^/Na^+^ and Cl^−^/Li^+^ for PVDF-DETA4 reached 0.9516 and 0.955, respectively. Those factors directly confirmed the anion-exchange behavior of investigated PVDF-DETA4 membrane. Considering the PSO results, the R^2^ correlation for all species was high and got over 0.9. This behavior suggested that both ion types could be accumulated on the membrane surfaces where the sorbate and functional groups had a main role in transportation. 

The Weber–Morris intraparticle diffusion model describes the influence of solid structure and its interaction with diffuse species on the transport rate across the membrane. Intraparticle diffusion is a transport process involving the movement of species from the bulk of the solution to the solid phase. The *K_ID_* describes the intraparticle diffusion coefficient in mg/g min^0.5^. As shown in Table 3, the highest value of *K_ID_* was obtained for chloride and nitrate transportation. On the other hand, based on Elovich fitting, the initial sorption rate was the highest for chloride and nitrates with a neglected small amount of sodium and lithium. In addition, the activation energy for chemisorption of chloride and nitrated were the smallest. Finally, the evaluation related to fitting data to adsorption isotherms was performed.

In this case, the Temkin and Harkin-Jura models were selected. Their fitting and specific parameters are summarized in Figure 9 and Table 3, respectively. The Temkin isotherms well fit all of the evaluated ions. The most crucial parameters are *A_T_* and *b_T_*, which indicate maximum binding energy and heat of adsorption, respectively. The most negligible binding energy was determined for chloride transportation, while for nitrated, sodium and lithium *A_T_* reach around 480 L/g. Significant differences are observed for the heat of adsorption. For chloride and nitrates, the *b_T_* was estimated at 128 and 223 kJ/mol, while sodium and lithium were at 1121 and 1090 kJ/mol. Hence, the energetical parameters required for sorption are over five times lower for anions than cations transportation. The last investigated adsorption isotherms were the Harkins–Jura model. The HJ model describes the multilayer adsorption and the heterogeneous pore distribution on the surface of the adsorbents. The HJ model exhibits a low R^2^ correlation to the experimental data for all evaluated ions. This means that the adsorption mechanism of chlorides, nitrates, sodium, and lithium are related to monolayer fulfilling and can be linked with adsorption on the surface without pores.

Considering the presented experimental data and analysis of adsorption kinetics and isotherms, it can be concluded that the PVDF-DETA4 exhibits a significant behavior for transportation anions like chloride and nitrates compared to sodium and lithium cations. Moreover, the developed anion exchange membrane enhances the chloride transportation of nitrates.

## 4. Conclusions

The presented research is related to preparing anion exchange membranes that can be used for concentration salts during the desorption step. The following outputs can be delivered:

- The modification of PVDF films by DETA runs according to Michael addition reaction and leads to the creation excellent anion exchange membrane with a high amount of chloride anions transportation.
- The best chemical and transportation properties were detected for PVDF modified by DETA by four days.
- The PVDF-DETA4 membrane is suitable to block the co-ions effect during the desorption step and allow to reach the SAC around 30 mg/g.
- The PVDF-DETA4 membrane allows performing the desorption step with 90% of efficiency.
- By applying the PVDF-DETA4 membrane, it is possible to concentrate the LiCl with four times the factor.
- Based on adsorption kinetics and isotherms, the PVDF-DETA4 exhibits the enhanced transportation of chlorides compared to nitrates, sodium, and lithium cations, which directly state the anion exchange ability of the developed membrane.

## Figures and Tables

**Table 1:** Parameters of the HCDI process.

General Parameters of HCDI			
A_cell_	Electrode geometric surface area	36	cm^2^
Φ_v_	Water flow rate	6	dm^3^/h
T	Temperature	25	°C
L_ch_	Thickness flow channel (100% open)	200	µm
M	Number of electrodes calls	1	pair
L_el_	Electrode thickness	80	µm

**Table 2:** Comparison of other membrane processes for lithium concentration.

Process	Type of Membrane	The Ratio of Li Concentration Increasing [%]	Time of Process [h]	Rate of Concentration [g/h]	Ref.
Forward osmosis	CTA membrane	410	30	0.41	[30]
Forward osmosis	Composite PVDF membrane	486	550	0.32	[31]
Pervaporation	PP/GO membrane	385	120	0.0035	[32]
Dialysis	CTA/Cyanex 923 and LIX-54-100	387	30	0.21	[33]
HCDI	PVDF-EDA24	700	1.6	0.18	[34]
HCDI	PVDF-DETA4	400	0.16	0.53	This study

**Table 3:** List of calculated parameters for PVDF-DETA4 membrane.

Model		Chlorides	Nitrates	Sodium	Lithium
Adsorption Kinetics					
PFO	*q_m_* (mg/g)	121.4	40.4	2.20	3.50
	*k*_1_	0.0062	0.0014	0.001	0.0016
	R^2^	0.963	0.943	0.658	0.888
PSO	*q_m_* (mg/g)	123.8	73.5	4.76	5.37
	*k*_2_	0.0004	0.0012	0.2756	0.198
	R^2^	0.968	0.909	0.987	0.934
WM	*K_ID_*	5.55	3.26	0.177	0.215
	*C*	−10.47	−6.06	1.19	0.65
	R^2^	0.984	0.969	0.844	0.943
Elovich	*α*	7.16	4.19	1.61	1.02
	*β*	0.058	0.098	1.53	1.40
	R^2^	0.751	0.748	0.908	0.828
Adsorption isotherms					
Temkin	*A_T_*	245.6	470.4	493.3	486.2
	*b_T_*	128.6	223.6	1121	1090
	R^2^	0.692	0.700	0.952	0.914
Harkin-Jura	*A_HJ_*	−0.128	−0.016	−0.004	−0.004
	*B_HJ_*	−0.150	−0.072	0.002	0.015
	R^2^	0.362	0.388	0.758	0.613

## Data Availability

Not applicable.

## References

[1] Hassanvand A., Chen G.Q., Webley P.A., Kentish S.E. (2017). Improvement of MCDI operation and design through experiment and modelling: Regeneration with brine and optimum residence time. Desalination.

[2] Ali A., Quist-Jensen C.A., Jørgensen M.K., Siekierka A., Christensen M.L., Bryjak M., Hélix-Nielsen C., Drioli E. (2021). A review of membrane crystallization, forward osmosis and membrane capacitive deionization for liquid mining. Resour. Conserv. Recycl..

[3] Siekierka A., Kujawa J., Kujawski W., Bryjak M. (2018). Lithium dedicated adsorbent for the preparation of electrodes useful in the ion pumping method. Sep. Purif. Technol..

[4] Siekierka A. (2019). Lithium iron manganese oxide as an adsorbent for capturing lithium ions in hybrid capacitive deionization with different electrical modes. Sep. Purif. Technol..

[5] Porada S., Shrivastava A., Bukowska P., Biesheuvel P.M., Smith K.C. (2017). Nickel Hexacyanoferrate Electrodes for Continuous Cation Intercalation Desalination of Brackish Water. Electrochim. Acta.

[6] Kim S., Lee J., Kim S., Kim S., Yoon J. (2018). Electrochemical Lithium Recovery with a LiMn_2_O_4_–Zinc Battery System using Zinc as a Negative Electrode. Energy Technol..

[7] Lee D.H., Ryu T., Shin J., Ryu J.C., Chung K.S., Kim Y.H. (2017). Selective lithium recovery from aqueous solution using a modified membrane capacitive deionization system. Hydrometallurgy.

[8] Lado J.J., Pérez-Roa R.E., Wouters J.J., Tejedor-Tejedor M.I., Federspill C., Ortiz J.M., Anderson M.A. (2017). Removal of nitrate by asymmetric capacitive deionization. Sep. Purif. Technol..

[9] Lee J., Kim S., Kim C., Yoon J. (2014). Hybrid capacitive deionization to enhance the desalination performance of capacitive techniques. Energy Environ. Sci..

[10] Siekierka A., Bryjak M., Wolska J. (2017). The use of activated carbon modified with polypyrrole as a supporting electrode for lithium ions adsorption in capacitive deionization. Desalin. Water Treat..

[11] Porada S., Zhao R., van der Wal A., Presser V., Biesheuvel P.M. (2013). Review on the science and technology of water desalination by capacitive deionization. Prog. Mater. Sci..

[12] Xin Y., Tian H., Guo C., Li X., Sun H., Wang P., Lin J., Wang S., Wang C. (2016). PVDF tactile sensors for detecting contact force and slip: A review. Ferroelectrics.

[13] Kang G.D., Cao Y.M. (2014). Application and modification of poly(vinylidene fluoride) (PVDF) membranes—A review. J. Memb. Sci..

[14] Dias A.J., McCarthy T.J. (1985). Dehydrofluorination of poly(vinylidene fluoride) in dimethylformamide solution: Synthesis of an operationally soluble semiconducting polymer. J. Polym. Sci. Polym. Chem. Ed..

[15] Poon T., Mundy B.P., Shattuck T.W. (2002). The Michael Reaction. J. Chem. Educ..

[16] Siekierka A., Wolska J., Bryjak M., Kujawski W. (2017). Anion exchange membranes in lithium extraction by means of capacitive deionization system. Desalin. Water Treat..

[17] Azizian S. (2004). Kinetic models of sorption: A theoretical analysis. J. Colloid Interface Sci..

[18] Simonin J.P. (2016). On the comparison of pseudo-first order and pseudo-second order rate laws in the modeling of adsorption kinetics. Chem. Eng. J..

[19] Siekierka A. (2019). Preparation of electrodes for hybrid capacitive deionization and its influence on the adsorption behaviour. Sep. Sci. Technol..

[20] Wu F.C., Tseng R.L., Juang R.S. (2009). Characteristics of Elovich equation used for the analysis of adsorption kinetics in dye-chitosan systems. Chem. Eng. J..

[21] Eriksson M., Lundström I., Ekedahl L.-G. (1997). A model of the Temkin isotherm behavior for hydrogen adsorption at Pd–SiO_2_ interfaces. J. Appl. Phys..

[22] Erdogan F.O. (2019). Freundlich, langmuir, temkin, dr and harkins-jura isotherm studies on the adsorption of CO_2_ on various porous adsorbents. Int. J. Chem. React. Eng..

[23] Kushwaha A.K., Gupta N., Chattopadhyaya M.C. (2014). Removal of cationic methylene blue and malachite green dyes from aqueous solution by waste materials of Daucus carota. J. Saudi Chem. Soc..

[24] Robati D. (2013). Pseudo-second-order kinetic equations for modeling adsorption systems for removal of lead ions using multi-walled carbon nanotube. J. Nanostruct. Chem..

[25] Riahi K., Chaabane S., Thayer B. (2017). Ben A kinetic modeling study of phosphate adsorption onto Phoenix dactylifera L. date palm fibers in batch mode. J. Saudi Chem. Soc..

[26] Chaudhry S.A., Zaidi Z., Siddiqui S.I. (2017). Isotherm, kinetic and thermodynamics of arsenic adsorption onto Iron-Zirconium Binary Oxide-Coated Sand (IZBOCS): Modelling and process optimization. J. Mol. Liq..

[27] Ayawei N., Ebelegi A.N., Wankasi D. (2017). Modelling and Interpretation of Adsorption Isotherms. J. Chem..

[28] Hernández-Monje D., Giraldo L., Moreno-Piraján J.C. (2018). Study of Hexane Adsorption on Activated Carbons with Differences in Their Surface Chemistry. Molecules.

[29] Taguet A., Ameduri B., Boutevin B. (2005). Crosslinking of Vinylidene Fluoride-Containing Fluoropolymers. Adv. Polym. Sci..

[30] Pham M.T., Nishihama S., Yoshizuka K. (2020). Concentration of lithium by forward osmosis. Hydrometallurgy.

[31] Wagh P., Islam S.Z., Deshmane V.G., Gangavarapu P., Poplawsky J., Yang G., Sacci R., Evans S.F., Mahajan S., Paranthaman M.P. (2020). Fabrication and Characterization of Composite Membranes for the Concentration of Lithium Containing Solutions Using Forward Osmosis. Adv. Sustain. Syst..

[32] Cha-umpong W., Li Q., Razmjou A., Chen V. (2021). Concentrating brine for lithium recovery using GO composite pervaporation membranes. Desalination.

[33] Paredes C., Rodríguez de San Miguel E. (2020). Selective lithium extraction and concentration from diluted alkaline aqueous media by a polymer inclusion membrane and application to seawater. Desalination.

[34] Siekierka A., Bryjak M. (2019). Novel anion exchange membrane for concentration of lithium salt in hybrid capacitive deionization. Desalination.

[35] Revellame E.D., Fortela D.L., Sharp W., Hernandez R., Zappi M.E. (2020). Adsorption kinetic modeling using pseudo-first order and pseudo-second order rate laws: A review. Clean. Eng. Technol..

